# A Remedy Proposed for the Evil of Substitution

**Published:** 1901-12

**Authors:** J. D. Williams

**Affiliations:** New York


### A Remedy Proposed for the Evil of Substitution.

BY J. D. WILLIAMS, M. D., NEW YORK.

There can be no subject of more importance to physicians than the violation of their confidence on the part of a dishonest dispensing druggist. Law will not make a dishonest man honest, but the right law properly executed will prevent a criminal's further infliction of injury upon society. The requirement of a license to all druggists who dispense drugs or medicines, revokable upon the licensee's being convicted of substituting any ingredient drug or medicine other than and in lieu or instead of that specified in the prescription, order or request in writing, of any physician, would go a long way to aid in the matter of honestly filling prescriptions. Let the medical societies induce their respective State legislatures to enact a law requiring such a license, with a simple and practical procedure for establishing the guilt and enforcing the penalty against infraction, and the practice of substitution would soon cease.

Let proceedings for revocation of license be before the court, board, or officer, empowered to issue the license, and be set in motion at the relation of either the board of health, a local medical society, or the purchaser upon whom the fraud and imposition had been done, or of the physician by whom the prescription or order was issued or given, or of any person, firm or corporation for whose brand or make of drug or medicine the substitution had been perpetrated. Let the licensing board, court, or officer be empowered to issue citations, subpœnas for witnesses, to administer oaths, and be given all other requisite powers for duly trying the issues and revoking the license of the guilty.—*Exchange.*

